# Supplementary figures and images for: Regulation of blood–brain barrier integrity by microbiome-associated methylamines and cognition by trimethylamine N-oxide
Source: Microbiome. 2021 Nov 27;9:235. doi: 10.1186/s40168-021-01181-z (PMC8626999; doi:10.1186/s40168-021-01181-z)

Supplementary Figure 1

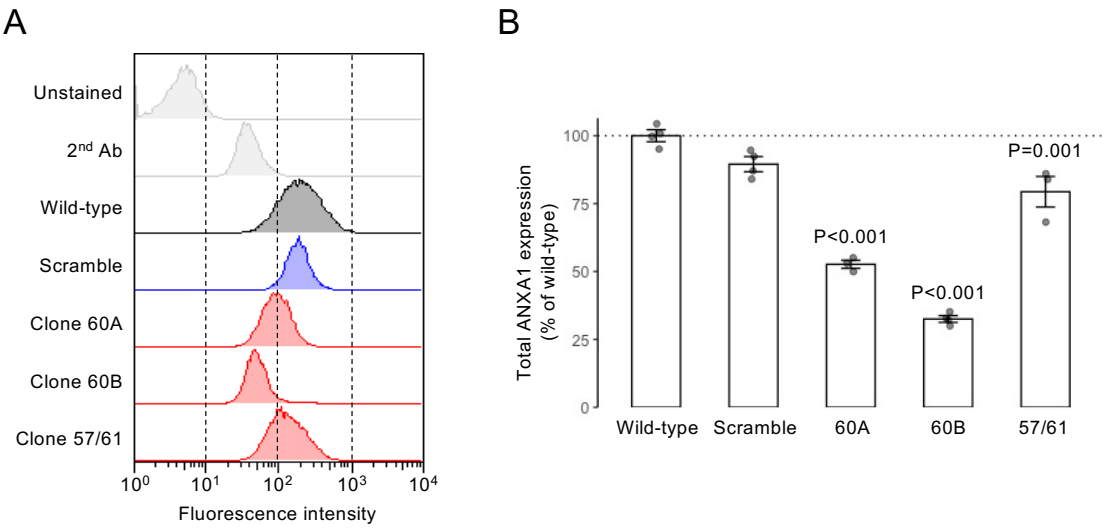

Supplementary Figure 2

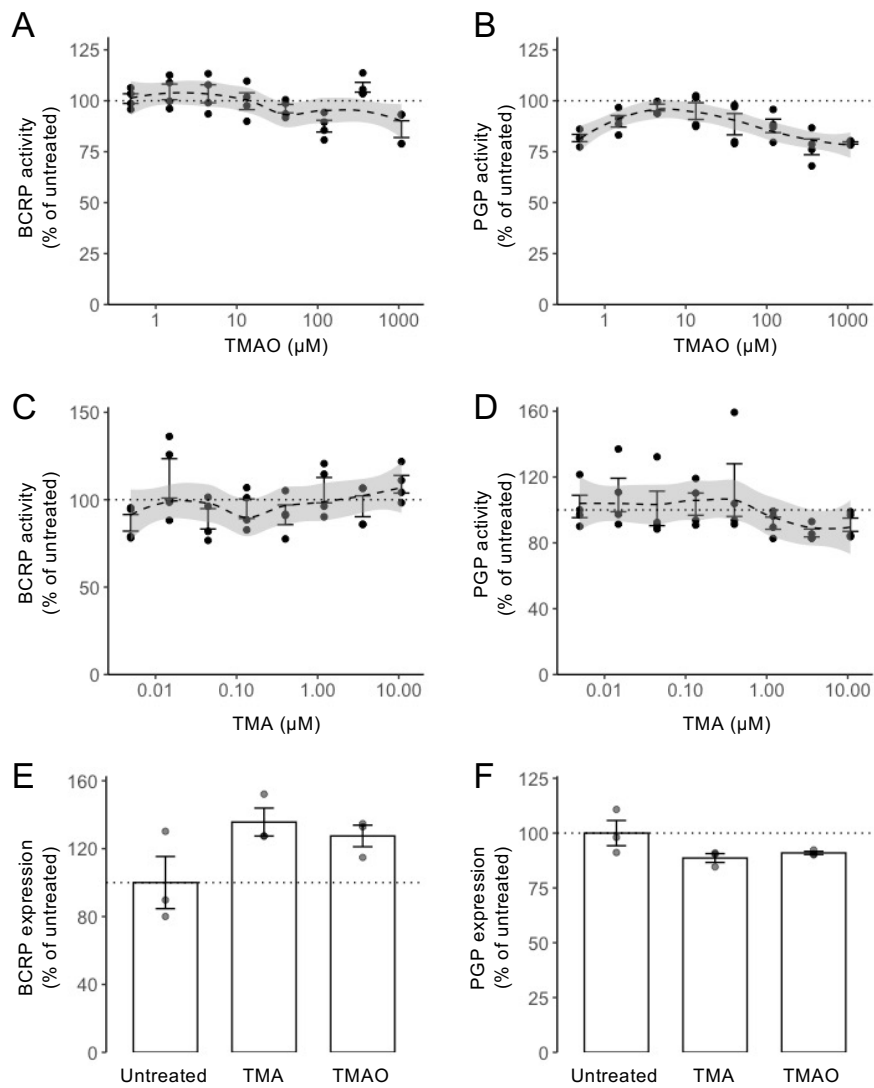

Supplementary Figure 3

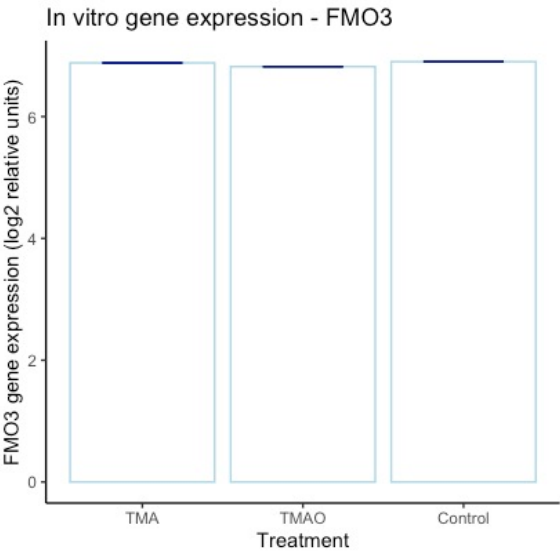

Supplementary Figure 4

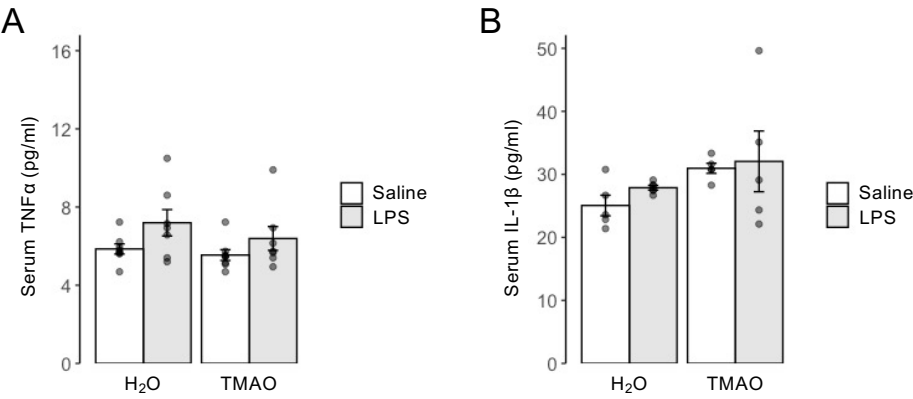

Supplement: Supplementary file 2 — Additional file 1: Supplementary Figures 1-4 [file 40168_2021_1181_MOESM2_ESM.pdf]
